# Bioavailability and Bioactivities of Polyphenols Eco Extracts from Coffee Grounds after In Vitro Digestion

**DOI:** 10.3390/foods9091281

**Published:** 2020-09-12

**Authors:** Emanuel Vamanu, Florentina Gatea, Diana Roxana Pelinescu

**Affiliations:** 1 Faculty of Biotechnology, University of Agronomic Science and Veterinary Medicine, 59 Marasti blvd, 1 district, 011464 Bucharest, Romania; 2 Centre of Bioanalysis, National Institute for Biological Sciences, 296 Spl. Independentei, 060031 Bucharest, Romania; florentina.gatea@incdsb.ro; 3 Department of Genetics, ICUB-Research Institute of the University of Bucharest, 36-46 Bd. M. Kogalniceanu, 5th District, 050107 Bucharest, Romania; drpelinescu@yahoo.com

**Keywords:** antioxidant activity, antiproliferative, chlorogenic acid, *Escherichia coli*, roasting degrees

## Abstract

Coffee grounds are a valuable source of bioactive compounds. In Romania, most of the amount obtained is lost through non-recovery; the rest is occasionally used as organic fertilizer. The coffee grounds were selected according to the roasting degree: blonde roasted (BR), medium roasted (MR), and dark roasted (DR). The study aimed to evaluate three extracts, obtained with a mixture of ethanol/water/acetic acid (50/49.5/0.5), depending on the roasting degree. The majority phenolic component, the antioxidant, and anti-inflammatory effect, as well as the role that gastrointestinal transit had on the bioavailability of bioactive compounds were determined. Chlorogenic acid was inversely proportional to the roasting degree. BR showed the best correlation between antioxidant and anti-inflammatory activities in vitro/in vivo. The antiproliferative capacity of the extracts determined an inhibitory effect on the tumor cells. Antimicrobial activities, relevant in the control of type 2 diabetes, were exerted through the inhibition of microbial strains (*Escherichia coli*). Following gastric digestion, BR demonstrated a maximum loss of 20% in the stomach. The recovery of coffee grounds depended on the pattern of functional compounds and the bioavailability of the main component, chlorogenic acid.

## 1. Introduction

Arabica coffee is the most widely used variety, being used in most coffee chains in the European Union. The choice is generally made based on the flavor, and the acceptability is influenced by the degree of roasting of the beans [1]. It influences both the consumption and the level of bioactive compounds contained in the resulting coffee grounds [2]. Coffee grounds have a variety of possible uses at the industrial level, most of them based on the fact that it is of non-edible substrates [3].

A large number of studies (meta-analyses) have been conducted to demonstrate the association between coffee consumption and health effects [4]. These have confirmed a reduction in the incidence of factors that lead to chronic conditions caused by oxidative stress [5]. A cohort study has shown that coffee consumption is inversely correlated with increased mortality in some risk groups. Thus, moderate consumption could be considered to lead to a reduction in cardiovascular risk [6]. In general, the pharmaco-nutrition approach of coffee is based solely on coffee consumption and not on the by-products (coffee grounds). The level of coffee consumed daily influences bioavailability and thus the physiological response is due to daily habits [7]. These may explain why certain population groups have a low incidence of developing the chronic degenerative disease [8], regardless of genetic heritage. The degree of roasting influences the total polyphenolic content of the coffee consumed. The positive effects on human health are associated with the presence of chlorogenic acid, with the storage and grinding time after roasting [9]. Thus, the residues obtained from the use of coffee beans (*Coffea arabica* L.) have a high potential for extraction of functional compounds with antioxidant, anti-inflammatory, and antiproliferative properties [10].

Some of the most important compounds are chlorogenic acids and their derivatives, which are responsible for protection against oxidative stress [11]. The effect is assumed to be due to the high bioavailability after green coffee consumption [12]. The coffee grounds contain this phenolic acid as a majority compound, in addition to gallic and protocatechuic acids. These compounds are also responsible for the in vitro expression of the antioxidant properties demonstrated by the analysis of the extracts [13]. Thus, no clear data are known about the use of these components in human health (coffee grounds valorization). To be used in obtaining functional products (supplements, for example), a bioavailability analysis is needed, which shows the metabolism and the effects on the interaction with the fluids in the human digestive tract [14]. An easy way to determine the impact of the metabolization process on the bioactive components is the use of in vitro simulators [15]. Through such studies, it is possible to determine the stability of the main elements that express the bioactivity after their assimilation until the interaction with the human microbiota [16]. The purpose of the present research was to identify the phenolic pattern to determine the antioxidant, anti-inflammatory, and antiproliferative effects of the extracts from the coffee grounds as a result of the three degrees of roast. Relative bioavailability, in vitro, after the gastrointestinal digestion process, was also determined.

## 2. Materials and Methods

### 2.1. Chemicals

The reagents of analytical grade (purity > 98%) were purchased from Agilent Technologies (Santa Clara, CA, USA). The filtration of solvents (Merck, Darmstadt, Germany) and solutions was realized through 0.2 µm membranes (Millipore, Bedford, MA, USA). All culture media were purchased from Oxoid Ltd. (Hampshire, UK).

### 2.2. Samples and Extraction Process

Samples—coffee grounds (based on the roasting degree [17]; Starbucks, Romania; 100% whole bean Arabica coffee) were collected after the process of coffee production at Philips HD8829/09 (Amsterdam, The Netherlands). The substrate drying was performed in a ventilated incubator, at 40 °C, for 24 h and kept in the dark [18]. The extraction was realized in Duran bottles (volume 1.00 L; 24 h, at room temperature, stirring 100 rpm) at a concentration of 1% coffee grounds with the solvent: ethanol/water/acetic acid = 50/49.5/0.5 [19]. Following extraction process, the mixture was filtered (under vacuum) using Whatman filter paper no. 1. The extracts were mixed with 7% maltodextrin and freeze-dried after complete homogenization. The dried extracts dissolved in 50% (*v*/*v*) ethanol were submitted to a series of dilutions ranging from 0.1 to 3%. The freeze-drying was realized in aseptic conditions to eliminate other interactions [20].

### 2.3. Quantification of Polyphenolic Compounds by *Capillary Zone Electrophoresis* (CZE)

In order to separate the polyphenols, a CZE technique was used. The separations were performed using Agilent 6100 CE instrument with diode array detector (DAD), with a CE capillary (Agilent Technologies, Ratingen, Germany), bare fused silica, (ID 50 μm, 72 cm effective length). The separation conditions were: voltage 30 kV, cassette capillary temperature 30 °C, hydrodynamic injection for sample and standards (35 mbar/12 s). The migration buffer (BGE) consisted of 45 mM sodium tetraborate, 0.9 mM sodium dodecyl sulfate (SDS) at pH = 9.35. Between migrations, the capillary was washed with 0.1M NaOH and water one minute, each followed by 2 min of washing with BGE. Data were processed using the ChemStation program. The standards and samples solutions were prepared and diluted in methanol. The identification of the compounds was made by standard addition and by comparing the retention times at λ = 280 nm (Figure S1) [21,22].

### 2.4. In Vitro Bioactivity Assays

The antiradical potential was determined using the DPPH (2,2-diphenyl-1-picrylhydrazyl) radicals according to the spectrophotometric protocol. The readings of the optical density of the reaction mixtures were performed at 517 nm (Helios, Thermo Fisher Scientific, Waltham, MA, USA) were also required. The expression of antiradical potential was in terms of the effective concentration (EC50); the extract concentration (µg/mL) needed to inhibit the DPPH activity by 50% after at least 30 min [20]. Ascorbic acid and TBHQ (tert-Butylhydroquinone)were positive controls.

The determination of reduction power was realized spectrophotometrically, at 700 nm (Helios, Thermo Fisher Scientific, Waltham, MA, USA). The results were expressed as the value of the extract concentration (µg/mL) required to obtain an optical density of 0.5, at the same wavelength [20]. Ascorbic acid and TBHQ were positive controls.

Inhibition of lipid peroxidation was determined by using egg yolk extract, at 532 nm (Helios, Thermo Fisher Scientific, Waltham, MA, USA). The inhibition of lipid peroxidation was indicated as the effective concentration (EC50); the extract concentration (µg/mL) required to inhibit the 50% after boiling at 95 °C and cooling for 30 min [23]. Ascorbic acid and TBHQ were positive controls.

Inhibition of protein denaturation was determined by using bovine albumin 1%, at 600 nm (Helios, Thermo Fisher Scientific, Waltham, MA, USA). The inhibition of protein denaturation was presented in terms of the effective concentration (EC50); the extract concentration (µg/mL) needed to inhibit the 50% after boiling at 70 °C and cooling the reaction mixture [24]. Acetylsalicylic acid (AAS) and ketoprofen (DKP) were positive controls.

The inhibition of hemolysis was determined by using fresh blood obtained from healthy volunteer donors. The absorbance was read at 540 nm and effective concentration (EC50), was the extract concentration (µg/mL) necessary to inhibit 50%, after three h of incubation [25]. Acetylsalicylic acid (AAS) and ketoprofen (DKP) were positive controls.

#### In Vivo Antioxidant Activity

The modified protocol was based on the use of a strain of *Saccharomyces boulardii* from the collection of the Laboratory of Pharmaceutical Biotechnologies, Faculty of Biotechnologies, USAMV (University of Agronomic Science and Veterinary Medicine) Bucharest. The viability analysis required to calculate the critical point was performed using peptone yeast extract agar (g/L; yeast extract 5g, streptomycin sulfate, 0.03 g, soya peptone, 10 g, glucose, 40 g, chloramphenicol, 0.05 g, agar, 15 g; Merck KGaA, Darmstadt, Germany). The cross between the viability and mortality lines (different concentrations of H_2_O_2_ (%)—0.1, 0.3, 0.5, 1, 2) defined the critical concentration, and was expressed as a critical point (%). The untreated sample represented the control. The antioxidant potential in vivo was expressed as percentage after the bioactive compound assimilation [26]. 

### 2.5. In Vitro Antiproliferative Assay

The Vybrant^®^ MTT cell proliferation test kit (Thermo Fisher Scientific, USA) was used to assess the cytotoxic effect of the extracts by measuring the metabolic activity of HCT-8 cells (human ileocecal carcinoma—HCT-8 cell line, passage 43). HCT-8 cells were cultivated in RPMI 1640 (Lonza, Switzerland) provided with 10% FBS (fetal bovine serum) (Biochrom, Germany) at 37 °C, with 5% CO_2_ until they had reached to a 60% confluence. The medium was replaced with a fresh medium together with extracts in three different concentrations of 10%, 5%, and 0.1%. After 24 h, the HCT-8 cells were washed twice with PBS (phosphate buffered saline) and incubated for another 2.5 h with MTT solution. The dye was dissolved in DMSO, and the 96 plates were read at 540 nm using Synergy HTX (BioTek, Winooski, VT, USA). Cell viability was calculated according to the formula: % survival = (average experimental absorbance/average control absorbance) × 100 [27]. The 50% ethanol was control. 

### 2.6. Quantitative Antibacterial Assay by Minimum Inhibitory Concentration (MIC) Determination

The antimicrobial activity of the extracts was analyzed by the microdilution method, following M07-CLSI (Clinical Laboratory and Standards Institute, Annapolis Junction, MD, USA) recommendations. For this study, two Gram-positive strains (S. aureus ATCC BAA 1026, *S. aureus* 1004, MRSA phenotype, and two Gram-negative strains (*E. coli* ACTCC 25922; *E. coli* B11, ESBL phenotype) were selected. The multidrug-resistant (MDR) strains were isolated from wound infections and were previously characterized [28,29]. Ethanol 50% was used as control.

### 2.7. In Vitro Bioavailability Test

The modified in vitro GIS1 system and an adapted protocol were used for a more realistic representation of the release of bioactive compounds and for determining the stability of the extracts in the simulated environment [30]. A gelatin capsule (mean weight 0.505 ± 0.01 g extract, size 0; BSC, Wenzhou, China) was used to simulate the ingestion of a functional supplement. The capsules were added in the simulated medium [20]. The modified in vitro gastrointestinal digestion protocol [31] was performed in a 250 mL Duran square bottle of borosilicate glass and consisted of:

- The tested sample was solubilized in 0.9% NaCl (sterile solution). A volume of 50 mL was made, in which a gelatin capsule was dissolved. 10% of this mixture contained a solution simulating sterilized human saliva, with the formula (g/L): 10 g carboxymethyl cellulose, 0.625 g KCl, 0.059 g MgCl_2_, 0.166 g CaCl_2_, 0.804 g K_2_HPO_4_, 0.326 g KH_2_PO_4_, pH 7—oral phase.
- A total of 20 mL of simulated gastric juice was added (pepsin 3 g/L, 3 g/L mucin were dissolved in 0.9% NaCl).
- The pH was adjusted to 2 with 1N HCl with a Behrotest peristaltic pump, Type PLP 33, flow rate 0.4–2.0 L/h and kept for 1.5–2 h—Gastric phase.
- It was supplemented with 25 mL 0.9% NaCl in which 7 mg/mL pancreatin and 5 mg/mL bile salts were dissolved.
- The pH was adjusted to 7.5 with 1M Na_2_CO_3_, with a Behrotest peristaltic pump, Type PLP 33, flow 0.4–2.0 L/h and kept for 2–4 h—Intestinal phase;
- At the end of each phase, a volume of 10 mL was collected and analyzed.

The bioavailability index was calculated by the formula: (quantity of presented phenolics/quantity of total phenolics) × 100 [26], where the quantity of presented/total phenolics was estimated based on the method detailed in the same paper.

#### In Vivo Bioavailability Test

The bioavailability index in vivo was determined through a modified method, by using the same strain mentioned at 2.4.1, in the same culture conditions. Chlorogenic acid (1 mg/mL) was the control, as a major phenolic compound. The culture medium without agar was used, and the extract (1 mg/mL), was added after filtration with sterile Millipore membrane (0.45 µm). The bioavailability index (BI) was calculated according to the formula: (quantity of absorbed phenolics/quantity of total phenolics) × 100. The quantity of absorbed phenolics was evaluated after the freeze-drying of the biomass. Total phenolics were assessed by the spectrophotometric method [26].

### 2.8. Statistical Analysis

All the parameters investigated were evaluated in triplicate, with the results expressed as the mean ± standard deviation (SD). The IBM SPSS Statistics 23 software package (IBM Corporation, Armonk, NY, USA) was used to calculate the mean and SD values. The significance level was: significant—*p* ≤ 0.05; very significant—*p* ≤ 0.01; and highly significant—*p* ≤ 0.001 using the normal distribution of the variables. The differences were assessed by ANOVA followed by a Tukey post hoc analysis. The analysis and correlation of the experimental data were performed by the IBM SPSS Statistics software package (IBM Corporation, Armonk, NY, USA) [20].

## 3. Results

### 3.1. Phenolic Compounds from Coffee Grounds Eco Extracts

The use of the coffee grounds for the extraction of biologically active compounds was presented in Table 1. The results showed that roasting degrees also influenced the extraction efficiency. For MR and DR there was a loss of the amount of bioactive compounds, up to half, compared to BR. The increase in roasting from MR to DR did not significantly influence the amounts of bioactive compounds. They varied on average by 15%, while BR showed an increase in the main polyphenol carboxylic acids (cinnamic and chlorogenic acids) by about 51%. Caffeic acid, one of the most common phenolic acids from coffee, was identified only in a small amount of 4.18 ± 0.51 µg/mL. Increasing the roasting degree halved the amount determined for MR and was missing in the case of DR. Ferulic acid was identified only in the case of BR, which corresponded to the increased extraction yield and demonstrated the biological value of such a product. Moreover, the obtained electropherograms confirmed the presence, in negligible quantities, of catechin-like compounds (data not shown). Their variation depended directly on the roasting degrees of the substrate but did not influence the final phenolic profile in the extracts.

### 3.2. Biologic Activities of Coffee Grounds Eco Extracts

In vitro anti-inflammatory and antioxidant activities of the coffee extracts, based on roasted degrees, were presented in Table 2. Large differences were obtained between the three extracts, but it turned out that BR showed the best correlation between antioxidant activity and anti-inflammatory, in vitro. The EC50 value was very close to at least one control used (AA with 1.00 ± 0.07 mg/mL). In particular, MR demonstrated the lowest EC50 value when inhibiting erythrocyte hemolysis, 1.25 ± 0.16 mg/mL. An insignificant difference was calculated between MR and DR for the same method, showing specificity at the roasting degree of the sample. The ascending order of EC50, BR < MR < DR, was the same for all methods used in the evaluation of the antioxidant potential. In contrast, for the anti-inflammatory activity, the ascending order of EC50 showed specificity to the chosen method and demonstrated an influence of the amount and/or presence of bioactive compounds. The color of the freeze-dried extracts did not influence the results because the presence of the maltodextrin in the process provided high stability.

**Figure 1:**
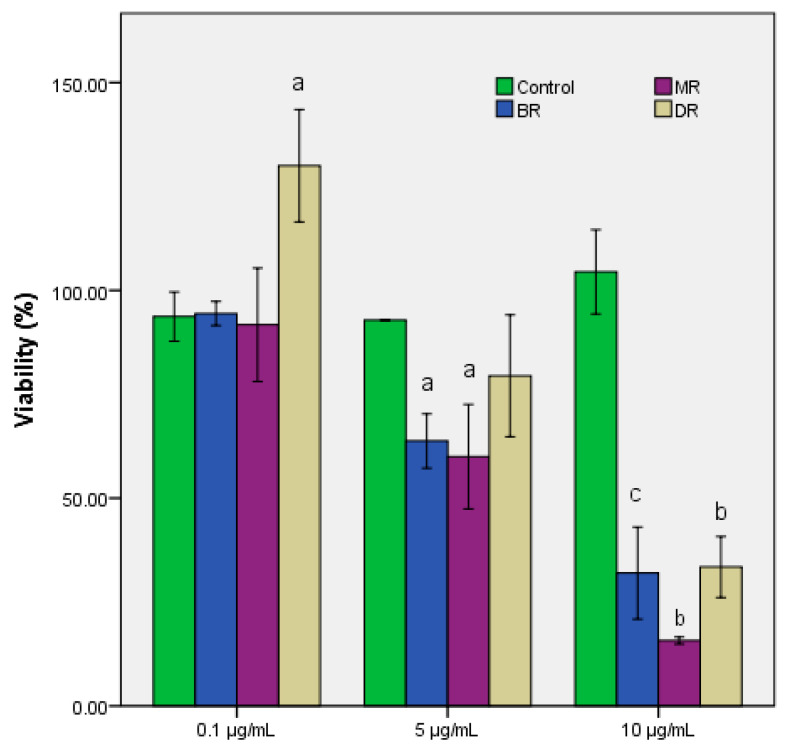
HCT-8 cell viability (% survival) after 24 h of incubation with the coffee grounds extracts based on roasting degrees. Mean values ± S.D.; different letters represent significant statistical differences (control vs. samples; *p* ≤ 0.05), *n* = 3.

The antiproliferative capacity of the extracts proved an inhibitory effect on the tumor cells (HCT-8 cells; Figure 1), at a concentration of 10%, the cell viability decreased between 65–85%, more than the control sample. At a level of 5%, BR and MR samples have reduced cell viability with almost 40%, while the DR sample had just a low effect. Instead, the control sample maintained the same antiproliferative effect. At a 0.1% concentration, BR and MR samples did not affect instead DR sample stimulate cell proliferation.

The MR extract had high antimicrobial activity against all microbial strains (Table 3), while the DR sample determined an increase of MIC with 50% on Enterobacteriaceae strains. Instead, BR extract exerted no antimicrobial activity against microbial strains. MR extract reduced the MIC of the MRSA 1004 strain from 25% to 6.25%, exercising a high antimicrobial activity. 

### 3.3. In Vivo Antioxidant Activity and Bioavailability

**Figure 2:**
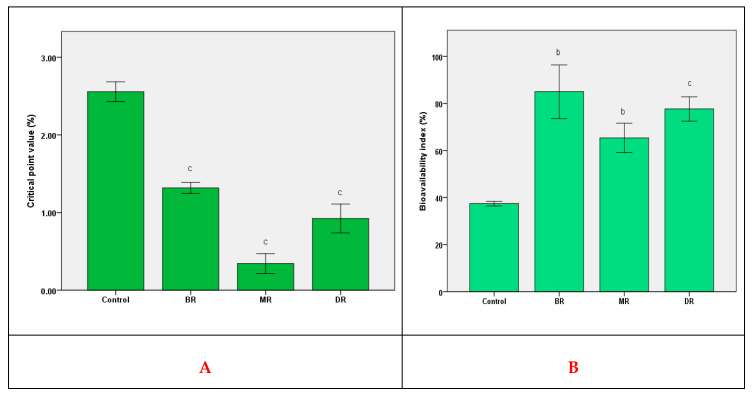
In vivo bioavailability (**right - B**) and antioxidant activity (**left - A**) of the coffee grounds extracts based on roasting degrees. Mean values ± S.D.; different letters represent significant statistical differences (control vs. samples; *p* ≤ 0.05), *n* = 3.

BR extract showed a maximum value of BI, and the difference compared to the other two extracts was between 10–25%. This has demonstrated the importance of pattern stability of functional compounds. Compared to a previous study [26], other compounds (like flavonoids) in the in vivo bioavailability process using *S. boulardii* strain were also active for coffee grounds extracts (Figure 2; Table 1).

The in vivo antioxidant activity (Figure 2) was directly proportional to the chlorogenic acid content as the primary compound. Based on the value of the critical point, its increase for BR was ≈60% higher than MR, 0.34 ± 0.04%. The results showed the assimilation of a different amount of compounds, according to the data presented in Table 1. Thus, the exo-protection to the action of reactive oxygen species was determined by the presence of chlorogenic acid, the rest of the compounds not directly influencing antioxidant activity in vivo.

### 3.4. Effect of In Vitro Gastrointestinal Digestion

**Figure 3:**
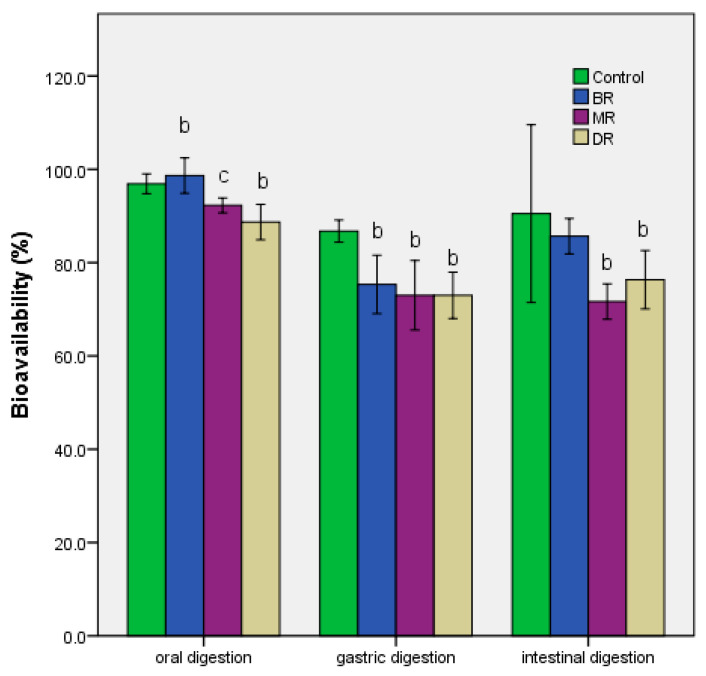
Bioavailability of phenolic compounds of the coffee grounds extracts based on roasting degrees. Mean values ± S.D.; different letters represent significant statistical differences (control vs. samples; *p* ≤ 0.05), *n* = 3.

The determination of bioavailability after the three stages of gastrointestinal digestion was presented in Figure 3. The value of this index decreased the most in the case of the second stage—the gastric passage. In the first stage, the differences between the extracts corresponded to the level of bioactive compounds (Table 1), and the loss demonstrated the impact that digestion had on the stability of functionality when administered in vivo [32]. Chlorogenic acid had high stability, which reduced the effect of oral digestion, *p* ≤ 0.01. The rest of the compounds showed low stability, with losses of up to 20%, a process continued during the second stage of digestion in vitro (Figure 3). The two hours of gastric digestion showed a reduction of up to 20%, even in the case of BR. In contrast, the addition of pancreatin and bile salts led to an increase in bioavailability. Finally, the *in vitro* digestion process resulted in losses of between 10–20% compared to undigested samples.

## 4. Discussions

Intestinal absorption of the active compound from a biopharmaceutical (drug and/or functional supplement) is a process that depends on several factors [33]. In addition to the physiological factors that influence the absorption, specific to each person, the metabolism in the gastrointestinal tract demonstrated efficiency in vivo [18]. Thus, the modified variant of GIS1 was a useful way to evaluate bioavailability vs. bioactivity rate of some bioactive molecules after human gastrointestinal transit [34], which had a limiting effect. In this respect, the bioavailability of the main component (chlorogenic acid) showed that the biopharmaceutical value of BR was high. The bioavailability of chlorogenic acid was different depending on the substrate, demonstrating that the presence of other components influenced the whole process and bioactivity [12].

In the case of the presented study, the relationship between the roasting degrees of coffee and the different activities determined in vitro was apparent. The extractive yield was one that directly influenced bioavailability. For coffee grounds, the roasting process was of essential importance in the characterization of a substrate [35]. Increasing the roasting degrees decreased the extractive yield, bioavailability, and functional significance of the substrate (Table 1). The results were a confirmation of recent studies that have shown that low roasting temperature was an essential condition for the valorization of coffee grounds [2] in the biopharmaceutical industry.

The main limiting factor was intestinal digestion (Figure 3), where the loss of stability of the main components (like caffeic acid) influenced the bioavailability process [36]. For other compounds, such as cinnamic acid, intestinal digestion proved a low antioxidant activity because of sensibility at alkaline pH that influenced the structural organization [37]. The low stability at the intestinal level was determined for isoquercitrin in a previous study [38]. For other compounds, like kaempferol, the presence at the intestinal level not determined a decrease of cell proliferation (DR sample), which was in contrast with past study (inhibition of proliferation in A549 cells) [39].

Thus, all three types of coffee grounds have demonstrated a high biological value, although they are a by-product of coffee. It was noted that the increase in roasting was inversely proportional to the bioactivity and bioavailability it expressed. Although there were differences (antimicrobial activity—Table 3), they proved that the presence of a single compound was not the critical point of bioactivity. This in vitro result represented an effect of the pattern of functional compounds that left a unique biofunctional imprint. The antimicrobial effect that was exerted on several levels (direct inhibition or disturbance of cellular functions [40]) was determined by the presence of certain compounds that imprinted a behavior and modulated the biological response. The pharmacological value of the extracts was a result of the convergence of several factors [41].

To evaluate the bioavailability in vivo, it was found that chlorogenic acid is the main compound on which the antioxidant potential expressed by the critical point value depends [26]. The determined differences were the result of the interaction with the rest of the compounds present that determined the BI value, compared to—the chlorogenic acid, the control. Compared to the in vitro test, there was a correlation with the results presented at the intestinal level. This study demonstrated that in vitro simulations provide an accurate characterization of functional products by the possibility of correlating in vitro results with in vivo ones without ethical limitations [42]. An essential aspect of the interpretation of the results was the use of the majority component as a control. The use of a eukaryotic organism was the key point in the validation of in vitro data and the interpretation of the response to the interaction with oxidative stress and the reduction of tumor cell proliferation [43].

This study showed that BR had the highest biopharmaceutical potential, an aspect that can be exploited by future research. This behavior was determined by the bioavailability of chlorogenic acid in gastrointestinal transit, compared to other types of coffee grounds [44]. The high, unabsorbed percentage has a significant pharmaceutical value, and will serve as a substrate for the modulation of the human microbiota [45]. It is assumed that it will show a bifidogenic effect, which will stimulate the synthesis of short-chain fatty acids and reduce the number of *Escherichia coli* strains [10]. With the balancing of the *Firmicutes* and *Bacteroides* ratio, it will be able to play a significant role in the formulation of products with a functional, protective role in the onset of type 2 diabetes, starting from a gradual modulation of the pattern of the human microbiota [46]. Instead, a limitation could result from the non-standardization of the substrate (coffee grounds) that can be recovered, but also from the germination of pathogenic fungal strains that limit the degree of valorization.

## 5. Conclusions

The data presented showed that coffee grounds could be valorized due to the pattern of functional compounds and the bioavailability in vitro and in vivo of the main component, chlorogenic acid. These aspects are correlated with the degree of roasting of the substrate and could have relevant physiological effects on human health. Thus, the colon microbiota has access to a different pattern of functional compounds that directly influence the bioactivity in the human colon. Research may be a starting point for studies showing that a coffee roasting degree can be beneficial in modulating the microbial pattern. Moreover, the modulation of metabolic activity, which determines the synthesis of the main SCFA, can be correlated with the ratio of bioactive compounds from BR, as a factor in maintaining homeostasis.

## Figures and Tables

**Table 1:** Quantification of phenolic compounds from (µg/mL) the coffee grounds extracts based on roasting degrees.

Compound (µg/mL)	BR	MR	DR
Caffeic Acid	4.18 ± 0.51 ^b^	2.04 ± 0.18 ^c^	nd
Cinnamic Acid	150.95 ± 2.08 ^a^	72.59 ± 0.23 ^b^	82.08 ± 2.84 ^a^
Chlorogenic Acid	135.07 ± 2.04 ^a^	47.75 ± 0.38 ^b^	56.67 ± 1.32 ^b^
Ferulic Acid	13.5 ± 0.38 ^c^	nd	nd
Coumaric Acid	13.98 ± 0.22 ^c^	4.56 ± 0.72 ^a^	4.67 ± 0.86 ^b^
Quercetol	11.82 ± 1.00 ^a^	4.26 ± 0.41 ^b^	4.47 ± 0.36 ^c^
Kaempferol	2.12 ± 0.22 ^c^	2.24 ± 0.17 ^c^	6.84 ± 0.56 ^b^
Isoquercitrin	8.28 ± 0.54 ^b^	6.86 ± 0.49 ^b^	22.69 ± 1.38 ^a^

Mean values ± S.D.; nd: non-detected; different letters indicate statistically significant differences (*p* < 0.05) between roasting degrees. Blonde roasted (BR), medium roasted (MR), and dark roasted (DR).

**Table 2:** In vitro anti-inflammatory, and antioxidant activities of the coffee grounds extracts based on roasting degrees (EC50 value, mg/mL).

**Methods for Antioxidant Activity**	**BR**	**MR**	**DR**	**AA**	**TBHQ**
Inhibition of lipid peroxidation	1.07 ± 0.08 ^a/^*	1.35 ± 0.03 ^a/^**	1.78 ± 0.09 ^b/^***	1.00 ± 0.07	0.75 ± 0.06
Reducing power	0.40 ± 0.003 ^a/^**	0.49 ± 0.002 ^b/^**	0.47 ± 0.01 **	0.64 ± 0.02	0.60 ± 0.01
DPPH scavenging activity	0.76 ± 0.11 ^c/^**	1.20 ± 0.10 ^c/^***	1.54 ± 0.05 ^c/^***	0.93 ± 0.07	0.45 ± 0.11
**Methods for Anti-Inflammatory Activity**	**BR**	**MR**	**DR**	**AAS**	**DKP**
Inhibition of protein denaturation	1.35 ± 0.09 ^b/^*	1.40 ± 0.02 ^c/^*	1.47 ± 0.01 ^b/^*	0.90 ± 0.10	1.20 ± 0.53
Inhibition of hemolysis	1.90 ± 0.03 ^b/^**	1.25 ± 0.16 ^b/^*	1.31 ± 0.06 ^c/^*	1.17 ± 0.08	1.37 ± 0.07

Mean values ± S.D.; different letters/asterisks represent significant statistical differences (AA/TBHQ or AAS/DKP vs. samples; *p* ≤ 0.05), *n* = 3.

**Table 3:** Minimum Inhibitory Concentration (MIC) (%) of the coffee grounds extracts based on roasting degrees against microbial strains.

Microbial Strains	BR	MR	DR	Control
*S. aureus* ATCC BAA 1026	12.5	6.25	12.5	12.5
*S. aureus* 1004	25	6.25	12.5	25
*E. coli* ACTCC 25922	12.5	6.25	25	12.5
*E. coli* B11	12.5	6.25	25	12.5

## Supplementary Materials

The following are available online at https://www.mdpi.com/2304-8158/9/9/1281/s1, Figure S1: Electropherograms for: (a) Standards (10 µg mL^−1^)—1-rutin, 2-naringenin, 3-isoquercitrin, 4-umbelliferone, 5-cinnamic acid, 6-chlorogenic acid, 7-syringic acid, 8-ferulic acid, 9-kaempferol, 10-luteolin, 11-coumaric acid, 12-quercetin, 13-rosmarinic acid, and 14-caffeic acid. (b) BR coffee grounds extract diluted two times.
