# Supplementary material for: Bioavailability and Bioactivities of Polyphenols Eco Extracts from Coffee Grounds after In Vitro Digestion
Source: Foods. 2020 Sep 12;9(9):1281. doi: 10.3390/foods9091281 (PMC7555697; doi:10.3390/foods9091281)
Supplement: Supplementary file 1 [file foods-09-01281-s001.pdf]

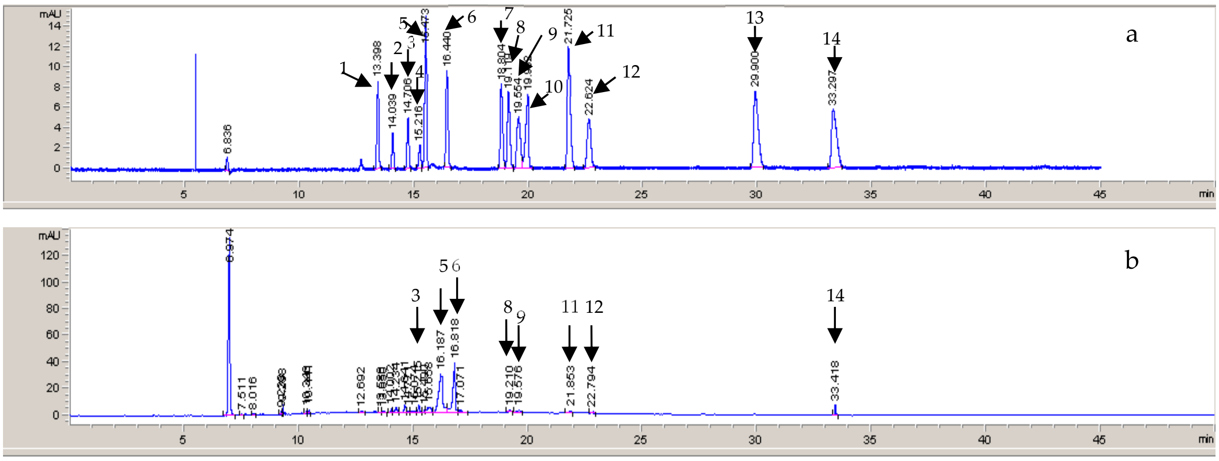

Figure S1. Electropherograms for: a) Standards (10μg mL^{-1}) - 1- rutin, 2-naringenin, 3-isoquercitrin, 4-umbelliferone, 5-cinnamic acid, 6-chlorogenic acid, 7-syringic acid, 8-ferulic acid, 9-kaempherol, 10-luteolin, 11-coumaric acid, 12-quercetin, 13- rosmarinic acid and 14-caffeic acid. b) BR coffee grounds extract diluted 2 times.
